# Associations between Indigenous Australian oral health literacy and self-reported oral health outcomes

**DOI:** 10.1186/1472-6831-10-3

**Published:** 2010-03-26

**Authors:** Eleanor J Parker, Lisa M Jamieson

**Affiliations:** 1 Australian Research Centre for Population Oral Health, The University of Adelaide, South Australia 5005, Australia

## Abstract

**Objectives:**

To determine oral health literacy (REALD-30) and oral health literacy-related outcome associations, and to calculate if oral health literacy-related outcomes are risk indicators for poor self-reported oral health among rural-dwelling Indigenous Australians.

**Methods:**

468 participants (aged 17-72 years, 63% female) completed a self-report questionnaire. REALD-30 and oral health literacy-related outcome associations were determined through bivariate analysis. Multivariate modelling was used to calculate risk indicators for poor self-reported oral health.

**Results:**

REALD-30 scores were lower among those who believed teeth should be infrequently brushed, believed cordial was good for teeth, did not own a toothbrush or owned a toothbrush but brushed irregularly. Tooth removal risk indicators included being older, problem-based dental attendance and believing cordial was good for teeth. Poor self-rated oral health risk indicators included being older, healthcare card ownership, difficulty paying dental bills, problem-based dental attendance, believing teeth should be brushed infrequently and irregular brushing. Perceived need for dental care risk indicators included being female and problem-based dental attendance. Perceived gum disease risk indicators included being older and irregular brushing. Feeling uncomfortable about oro-facial appearance risk indicators included problem-based dental attendance and irregular brushing. Food avoidance risk indicators were being female, difficulty paying dental bills, problem-based dental attendance and irregular brushing. Poor oral health-related quality of life risk indicators included difficulty paying dental bills and problem-based dental attendance.

**Conclusions:**

REALD-30 was significantly associated with oral health literacy-related outcomes. Oral health literacy-related outcomes were risk indicators for each of the poor self-reported oral health domains among this marginalised population.

## Background

Oral health is integral to overall health and wellbeing, with poor oral health and untreated oral conditions having a deleterious impact on quality of life [1]. Preventable and treatable oral diseases remain widespread, particularly amongst poor and underserved populations [2].

Indigenous Australians identify as being of Aboriginal and/or Torres Strait Islander descent, and represented 2.5% of the total Australian population in 2006. The median age is 21 years, compared with 37 years for the non-Indigenous population [3]. The majority of Indigenous Australians live outside major cities, with 43% living in regional and 25% in remote areas in 2006.

Indigenous Australians have poorer self-reported health and suffer a greater burden of disease than non-Indigenous Australians [3]. Indigenous adults accessing public dental services in Australia have higher levels of periodontal disease and fewer filled teeth, but greater numbers of missing teeth than non-Indigenous patients [4]. Indigenous children in Australia experience significantly higher levels of dental caries than their non-Indigenous counterparts [5,6] with greater levels of untreated disease and less preventive therapies [7].

Although recently gaining more attention, there has been little work in the field of oral health literacy or, more specifically, the impact of oral health literacy on oral health outcomes, amongst disadvantaged groups such as Indigenous Australians. Health literacy has been defined as "the degree to which individuals can obtain, process and understand the basic health information and services they need to make appropriate health decisions" [8]. In the oral health context, literacy can be considered as the skills necessary for people to understand the causes of poor oral health, to learn and adopt fundamental aspects of positive oral self-care behaviours, to communicate with oral health care providers, to place their names on dental treatment waiting lists or organise appointments, to find their way to the dental clinic, to fill out the necessary forms and to comply with any required regimes, including follow-up appointments and compliance with prescribed medication [9]. This definition addresses functional oral health literacy, encompassing knowledge as well as ability to use that knowledge in making appropriate oral health-related decisions. Oral health literacy, in this definition, encompasses far more than reading; it involves writing, numeracy, speaking, listening and 'understanding the system' [10]. It is suggested that the complexity of both verbal and written oral health communications create a significant barrier to improving oral health [2] and that oral health literacy is required in order to promote oral health and to prevent oral disease [1]. It has also been proposed that health literacy may be associated with barriers to accessing care, oral health behaviours such as prevention and to follow-up care [11].

**Figure 1:**
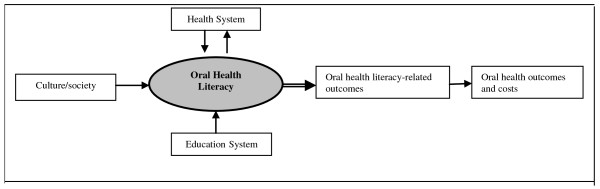
**Conceptual framework of oral health literacy and oral health literacy-related outcomes (modified from **[12]).

Although the precise relationship between literacy and oral health outcomes has not been established [1], one model that may be useful when conceptualising the interplay between oral health literacy, culture and society, the health system, the education system, and their collective role in determining oral health literacy-related outcomes and costs is outlined in Figure 1[12]. As depicted in the model, literacy is hypothesized as being one of many factors that influences oral health. The first step toward discerning the role of literacy in a multidimensional model of oral health is to therefore determine if literacy skills explain oral health disparities, or if disparities still exist among those with equivalent levels of literacy. Once the relationship between literacy and oral health (independent of education and other social determinants) is assessed, other factors in the explanatory model can be incorporated to see how they interact with oral health literacy. According to the model, such determinants include economics, cultural and other social factors, education and various aspects of the health system.

Word recognition tests demonstrate a strong correlation with general reading ability and reading comprehension [13], with evidence suggesting that if a person has difficulty pronouncing dental-related words, then that person may additionally have difficulty with comprehension; a higher order skill [14]. In the general health realm, those with limited health literacy skills are more likely to miss important preventative measures such as mammograms, Pap smears and influenza shots [15], and be late presenters to the health care system [16]. Low health-literate individuals often have chronic conditions and are less able to effectively manage them, for example, low-literate people with diabetes [17], asthma [18] or HIV/AIDS [19] have been shown to have less knowledge of their illness and its management than their more literate counterparts. Limited health literacy is associated with poor self-ratings of health [20], and an increase in preventable hospital admissions, with higher rates of hospitalisation and use of emergency services being reported among those with limited literacy [21]. Based on the Rapid Estimate of Adult Health Literacy in medicine (REALM), an instrument to measure dental health literacy (Rapid Estimate of Adult Literacy in Dentistry; REALD) was developed by Richman and colleagues [22]. A shortened version, REALD-30, was also developed and validated, with low REALD-30 scores being associated with poor oral health-related quality of life and poor self-rated oral health [23].

This study aims to contribute to an increased understanding of the impact of oral health literacy, and oral health literacy-related outcomes, on self-reported oral health among rural-dwelling Indigenous Australians. Specifically, the aims are: 1) to determine the relationship between oral health literacy, as assessed by REALD-30, and oral health literacy-related outcomes; defined in this study as oral health knowledge, oral health self-care and utilisation of dental services and; 2) to determine if oral health literacy-related outcomes are risk indicators for 7 domains of poor self-reported oral health.

## Methods

### Background

The authors had previously worked closely with the Indigenous community in the small regional town of Port Augusta, South Australia, Australia. In previous projects, focus groups had revealed themes of concern such as poor oral health systems navigation and a poor understanding of oral health information and health behaviours [24]. Following feedback from the community, this study was developed to investigate associations between oral health literacy and self-reported oral health outcomes.

### Study design

This was as a cross-sectional study of a convenience sample of Indigenous adults living in the Port Augusta region. Administration of a self-report questionnaire occurred during a one-week period [additional file 1].

### Recruitment

Recruitment techniques included word of mouth, attendance at health promotion sessions and community centres, the waiting room of the health service, interviews on radio, flyers, street stalls, home visits and Indigenous Health Worker contact. Where sessions had been more formally arranged by Indigenous Health Workers, morning and afternoon tea as well as transport was provided.

### Criteria

Participants needed to identify as being Indigenous, live in the Port Augusta region, be aged 17+ years and be able to understand and communicate in spoken English.

### Incentive

Participants received a $20 supermarket voucher upon completion of the questionnaire.

### Ethical approval

Ethics approval was granted by the Aboriginal Health Council of South Australia and the Human Research Ethics Committee of the University of Adelaide. Participants gave written informed consent before participating. Participants with limited reading ability had consent forms read to them.

### Self-reported questionnaire

Items included those used by the Australian Research Centre for Population Oral Health in other population-level surveys. The questionnaire was tested with five Indigenous adults and modified according to feedback received. With the exception of REALD-30, which required an interview, the questionnaire was administered through a combination of interview and self-complete approaches. The level of self-completion was determined by participants, with all questionnaires being reviewed by the interviewer to ensure completion. The questionnaire took approximately ten minutes to complete, and was completed in a number of settings including community halls, Indigenous resource centres, Pika Wiya Health Service, at a street stall outside the local supermarket, in people's homes and in schools.

### Dependent variables

Dependent variables were 7 domains of poor self-reported oral health. The 7 domains included: (1) having had one or more teeth extracted; (2) rating oral health as 'fair or poor'; (3) perceived need for fillings or extractions; (4) perceived gum disease; (5) feeling uncomfortable about appearance of teeth, mouth or false teeth; (6) having avoided eating some foods because of problems with teeth, mouth or false teeth and; (7) poor oral health related quality of life, as assessed by one or more OHIP-14 items rated 'very often' or 'fairly often' [25].

### Independent variables

Independent variables included demographic factors such as age and sex, socio-economic factors such as ownership of a means-tested Government-issued health care card, financial factors such as perceived difficulty paying a $100 dental bill, oral health literacy (REALD-30) and oral health literacy-related outcomes such as use of dental services (usual reason for seeing a dentist), oral health knowledge (number of times should brush teeth each day, is cordial good for teeth) and oral self-care (did brush teeth the previous day).

### Data analytic approach

Bivariate analyses were conducted to test the relationship between oral health literacy (REALD-30) and oral health-literacy related outcomes (dental service utilisation, oral health knowledge, oral self-care), as predicted by our theoretical model (Figure 1).

Univariate and bivariate distributions of the 7 dependent variables were determined. Correlation tests confirmed the existence of weak associations between independent variables in a given group (Pearson's correlation coefficient range 0.1-0.4), with no variables needing to be excluded due to collinearity. The high prevalence of the 7 domains of poor self-reported oral health meant that odds ratios were poor indicators of relative frequency, so prevalence ratios were determined using Poisson regression modelling [26]. Poisson regression analysis was used to derive adjusted estimates for the prevalence of the dependent variables. Guided by the theoretical model, exposure variables were classified into demographic, socio-economic, financial, oral health literacy and oral health literacy-related outcomes. The risk indicators significantly associated with poor self-reported oral health at a bivariate level were evaluated in multivariate Poisson regression models, based on the conceptual model (Figure 1). The regression models were constructed by removing covariates one at a time according to P-value size, with only values that remained statistically significant being presented in the final models. Data were analysed using SPSS 15.0 and Intercooled STATA 8.

## Results

Complete questionnaires were obtained from 468 participants, with an average age of 38 years (age range 17 to 72 years) and 63 percent female. The mean REALD-30 score was 15.0 (se = 0.36). Oral health literacy, as assessed by REALD-30, was lower among those who believed teeth should be brushed none or once daily, believed that cordial was good for teeth, did not own a toothbrush or owned a toothbrush but did not brush the previous day (Table 1).

**Table 1:** Associations between oral health literacy (REALD-30) and oral health literacy-related outcomes among Indigenous adults in Port Augusta; n = 468

Oral health literacy-related outcomes	Oral health literacy; mean reald-30 (se)
*Use of dental services*	
Reason for last dental visit	
Problem	15.3 (0.5)
Check-up	15.4 (0.7)
	
*Oral health knowledge*	
How many times do you think you should brush your teeth each day?	
None or once	12.4 (1.0)*
Twice or more	15.4 (0.4)
	
Do you think cordial is good or bad for teeth or gums?	
Good	11.8 (0.9)*
Bad	15.7 (0.4)
	
*Oral self-care*	
Do you own a toothbrush?	
Yes	15.9 (0.4)*
No	10.4 (0.9)
	
If yes, did you brush your teeth yesterday?	
Yes	16.0 (0.4)*
No	14.0 (1.0)

*P < 0.05

The prevalence of having had a tooth removed was higher among those aged 38+ years, those with low oral health literacy scores, who usually visited a dentist because of a problem and who believed cordial was good for teeth (Table 2). 'Fair or poor' self-rated oral health was higher among those aged 38+ years, males, those who owned a health care card, those reporting a lot of difficulty paying a $100 dental bill, problem-based dental attenders, those who believed teeth should be brushed none or once daily and those who did not brush teeth the previous day. Self-perceived need for fillings or extractions was higher among females, those with low oral health literacy scores and problem-based dental attenders. A higher prevalence of those who perceived they had gum disease were aged 38+ years, owned a health care card, reported difficulty paying a $100 dental bill, were problem-based dental attenders and did not brush teeth the previous day. Feeling uncomfortable about the appearance of one's teeth, mouth or dentures was higher among those aged 38+ years, those with low oral health literacy scores, problem-based dental attenders and those who did not brush teeth the previous day. Avoiding eating some foods because of problems with teeth, mouth or dentures was higher among those aged 38+ years, females, those reporting difficulty paying a $100 dental bill, those with low oral health literacy scores, problem-based dental attenders and those reporting that they did not brush the previous day. Poor oral health-related quality of life-as assessed by one or more OHIP-14 items reported 'very often' or 'fairly often'-was higher among those aged 38+ years, those reporting difficulty paying a $100 dental bill, those with low oral health literacy scores and problem-based dental attenders.

**Table 2:** Prevalence (%) of poor self-rated oral health outcomes among Aboriginal adults in Port Augusta by risk indicators; n = 468

	Have had teeth pulled out	Rate oral health as fair or poor	Need fillings or extractions	Think have gum disease	Uncomfortable about appearance of mouth	Avoid eating some foods	Poor oral health-related quality of life
*Total*	69.7	37.0	55.6	21.4	56.0	55.1	34.8
*Demographic*							
Age							
37 years or less	61.5*	26.9*	54.7	15.5*	51.3*	49.6*	29.9*
38 years or more	78.4	47.0	56.5	27.4	60.7	60.7	39.7
							
Sex							
Male	68.1	42.5*	49.1*	21.1	52.7	49.7*	31.1
Female	70.6	33.9	59.3	21.6	57.8	58.1	36.9
							
*Socio-economic*							
Health Care Card							
Yes	67.9	40.2*	55.3	23.2*	56.1	55.5	36.8
No	74.8	26.3	56.1	15.2	55.3	54.4	28.9
							
*Dental cost*							
Difficulty paying $100 dental bill							
None, hardly any, a little	67.9	29.1*	52.5	18.1*	54.5	51.2*	28.7*
A lot	71.7	45.5	59.1	25.1	57.6	59.4	41.5
							
*Oral health literacy*							
Mean REALD-30 (se)	13.8 (0.7)*	14.9 (0.5)	13.6 (0.5)*	14.9 (0.4)	13.8 (0.5)*	11.2 (0.5)*	14.0 (0.5)*
							
ORAL HEALTH LITERACY-RELATED OUTCOMES							
*Use of dental services*							
Usual reason for seeing dentist?							
Problem	83.9*	44.3*	66.6*	25.7*	66.0*	67.6*	42.7*
Check-up	47.5	19.4	38.2	15.4	37.1	34.7	19.4
							
*Oral health knowledge*							
Number times should brush teeth daily?							
None or once	69.3	46.2*	51.9	26.3	51.3	51.3	35.9
Twice or more	69.8	35.1	56.4	20.5	56.9	55.9	34.6
							
Is cordial good for teeth?							
Yes	80.0*	36.8	46.4	26.8	63.2	61.4	40.4
No	68.3	37.0	56.9	20.7	55.0	54.3	34.1
							
*Oral self-care*							
Did brush teeth yesterday?							
Yes	70.5	30.9*	55.7	17.3*	54.0*	52.3*	32.1
No	64.7	58.8	61.8	41.2	67.6	66.2	36.8

*P < 0.05

Risk indicators for having had one or more teeth removed included being aged 38 years or more, usually visiting a dentist because of a problem and believing that cordial was good for teeth (Table 3). Risk indicators for self-rated oral health as 'fair or poor' included being aged 38 years or more, ownership of a health care card, having a lot of difficulty paying a $100 dental bill, usually attending a dentist because of a problem, believing teeth should be brushed none or once daily and not brushing teeth the previous day. Risk indicators for perceived need for fillings or extractions included being female and usually visiting a dentist because of a problem. Risk indicators for the fourth poor self-reported oral health domain, perceived gum disease, included being aged 38 years or more and not brushing teeth the previous day. Risk indicators for feeling uncomfortable about the appearance of one's teeth, mouth or dentures included usually visiting a dentist because of a problem and not brushing teeth the previous day. Risk indicators for avoiding eating some foods were being female, having a lot of difficulty paying a $100 dental bill, usually visiting a dentist because of a problem and not brushing teeth the previous day. Risk indicators for the final domain of poor self-reported oral health, poor oral health-related quality of life, included a lot of difficulty paying a $100 dental bill and usually visiting a dentist because of a problem.

**Table 3:** Adjusted prevalence ratios for poor oral health outcomes among Aboriginal adults in Port Augusta; n = 468 (95% CI in parentheses)^†^

	Have had teeth pulled out	Rate oral health as fair or poor	Need fillings or extractions	Think have gum disease	Uncomfortable about appearance of mouth	Avoid eating some foods	Poor oral health-related quality of life
*Demographic*							
Age							
37 years or less	ref	ref	-	ref	-	-	-
38 years or more	1.09 (1.02-1.16)	1.13 (1.06-1.20)	-	1.07 (1.02-1.11)	-	-	-
							
Sex							
Male	-	-	ref	-	-	ref	-
Female	-	-	1.08 (1.01-1.15)	-	-	1.11 (1.04-1.19)	-
							
*Socio-economic*							
Health Care Card							
Yes	-	1.09 (1.02-1.18)	-	-	-	-	-
No	-	ref	-	-	-	-	-
							
*Dental cost*							
Difficulty paying $100 dental bill							
None, hardly any, a little	-	ref	-	-	-	ref	ref
A lot	-	1.11 (1.04-1.19)	-	-	-	1.09 (1.02-1.17)	1.47 (1.14-1.88)
							
ORAL HEALTH LITERACY-RELATED OUTCOMES							
*Use of dental services*							
Usual reason for seeing dentist?							
Problem	1.29 (1.20-2.38)	1.14 (1.07-1.23)	1.21 (1.14-1.30)	-	1.20 (1.12-1.28)	1.22 (1.14-1.30)	2.15 (1.47-3.15)
Check-up	ref	ref	ref	-	ref	ref	ref
							
*Oral health knowledge*							
Number times should brush teeth daily?							
None or once	-	1.11 (1.02-1.22)	-	-	-	-	-
Twice or more	-	ref	-	-	-	-	-
							
Is cordial good for teeth?							
Yes	1.09 (1.00-1.19)	-	-	-	-	-	-
No	ref	-	-	-	-	-	-
							
*Oral self-care*							
Did brush teeth yesterday?							
Yes	-	ref	-	ref	ref	ref	-
No	-	1.20 (1.11-1.31)	-	1.15 (1.06-1.24)	1.14 (1.04-1.26)	1.20 (1.11-1.31)	-

^†^All outcomes adjusted for risk indicators significant at a bivariate level, including REALD-30. Only risk indicators remaining statistically significant in the final multivariate models are presented

## Discussion

Based on a conceptual model, this study set out to determine if: a) oral health literacy, as assessed by REALD-30, was associated with the oral health literacy-related outcomes of dental service utilisation, oral health knowledge and oral self-care behaviour and; b) if oral health literacy-related outcomes were risk indicators for 7 domains of poor self-reported oral health among a convenience sample of rural-dwelling Indigenous Australians. REALD-30 was significantly associated with the oral health literacy-related outcomes. Consistent with our conceptual framework-that portrays oral health literacy as preceding oral health literacy-related outcomes in terms of oral health consequences-REALD-30 did not persist as a risk indicator for poor self-reported oral health in the multivariate models. However, at least one of the risk indicators for each of the poor self-reported oral health measures included oral health literacy-related outcomes.

Before examining our findings in greater detail, it is important to describe the study's shortcomings. First, the sample was one of convenience, meaning the findings cannot be considered to be representative of all Indigenous persons in Port Augusta. Due to the convenience nature of the recruitment strategies, the number of people who declined to participate was not recorded. Second, the design was cross-sectional, meaning there can be no assumptions of causality. Third, REALD-30 may not have been a realistic assessment of oral health literacy in our study population. The shortcomings of REALD-30 are acknowledged, particularly in that it measures word recognition only, that is, with no test of comprehension or function. However, there were few other validated instruments available to measure oral health literacy that were considered culturally acceptable to our Indigenous reference group. The Test of Functional Health Literacy in Dentistry (TOFHLiD) was developed in an attempt to measure broader aspects of oral health literacy, measuring reading comprehension as well as numerical ability [14]. This instrument was included in the initial questionnaire, but was removed after trialling with the Indigenous reference group, who identified a potential lack of acceptance within the community.

Shortcomings aside, the findings confirm that those with poorer oral health literacy, as measured by REALD-30, had poorer oral health knowledge and engaged in more harmful oral health literacy-related behaviours. The findings also indicate that, after adjusting for confounding, poor oral health literacy-related outcomes were risk indicators for 7 domains of poor self-reported oral health; which included items as far ranging as perceived need for dental care to oral health-related quality of life. The number of questionnaires completed was higher than anticipated, demonstrating that the project was embraced by the community, with many people involved and the majority of questionnaires implemented by Indigenous staff and community members. This survey has lead to further discussions with key members of the community, with ongoing discussions in relation to appropriate interventions, future research projects related to oral health, and development of an Indigenous Advisory Group for Indigenous oral health research in the region; important steps in the development of oral health research protocols that are owned and organised by the Indigenous groups of whom they intend to benefit.

The causal pathway between poor oral health literacy and poor oral health literacy-related outcomes-defined in our study as problem-based dental service utilisation, poor oral health knowledge and sub-optimal oral self-care behaviour-is both intuitive and supported by literature in the general health realm. For example, low health literacy has been associated with greater emergency visits to hospital [21], poorer knowledge regarding a chronic condition and its causes [18] and less-than-ideal self-care behaviour [15]. Our findings add evidence to the claim that literacy is one of the key ways in which individuals are able to process and act on information to improve their health outcomes and health care behaviours [27].

The associations between poor oral health literacy-related outcomes with the seven domains of poor self-rated oral health selected in this study are perhaps also intuitive, and again supported by literature in the general health realm. For example, limited health-related knowledge is a risk indicator for poor self-reported general health [28], and poor general self-care behaviour is a risk indicator for poor general health-related quality of life [29]. Given the evidence correlating poor self-reported oral health with poor clinical outcomes [30], our findings suggest that further investigation of the specific role of oral health literacy on oral health literacy-related outcomes and, in turn, the role of oral health literacy-related outcomes on various domains of poor self-rated oral health, warrants further investigation. This research is particularly relevant among Indigenous populations both in Australia and at an international level; groups who experience unacceptable levels of both dental disease and poor oral health-related quality of life, and cannot always access the care they require.

## Conclusions

In this convenience sample of Indigenous adults, oral health literacy was significantly associated with oral health literacy-related outcomes. In turn, oral health literacy-related outcomes were risk indicators for poor self-reported oral health. Further investigation is needed to better understand causal pathways and develop appropriate intervention strategies to improve oral health outcomes for Indigenous people.

## Competing interests

The authors declare that they have no competing interests.

## Authors' contributions

EJP drafted the manuscript. LMJ conducted the analysis and participated in the writing and completion of the manuscript. Both authors read and approved the final manuscript.

## Pre-publication history

The pre-publication history for this paper can be accessed here:

http://www.biomedcentral.com/1472-6831/10/3/prepub

## Supplementary Material

Additional file 1 **Aboriginal Oral Health Literacy Survey**. itemised survey questions pertaining to the analysis described in this paper. Click here for file
