# Supplementary material for: Associations between Indigenous Australian oral health literacy and self-reported oral health outcomes
Source: BMC Oral Health. 2010 Mar 26;10:3. doi: 10.1186/1472-6831-10-3 (PMC2859391; doi:10.1186/1472-6831-10-3)
Supplement: Additional file 1 — Aboriginal Oral Health Literacy Survey. itemised survey questions pertaining to the analysis described in this paper. [file 1472-6831-10-3-S1.DOC]

Aboriginal Oral Health Literacy Survey Questions: Appendix 1

Socio-demographic:

Date of birth

Sex

Indigenous status

Location: Pt Augusta? Other?

Highest qualification: primary school, high school. Trade or TAFE, University

Income: job? welfare?

Health care card?

Do you own a car?

Dental health status:

Do you have any of your own teeth left?

If no, do you have false teeth?

If yes, have you ever had any teeth pulled out?

If yes, how many?

Dental behavioural:

Have you seen a dentist before?

If yes, when did you last see a dentist? Less than one year ago? More than one year ago?

If yes, where did you last see a dentist? Public? Private?

If yes, what is your usual reason for seeing a dentist? Problem? Check-up?

Do you think you need to see a dentist? Yes? No?

Would you feel scared about going to the dentist? Yes? No?

Do you have a toothbrush? Yes? No?

If yes, did you brush your teeth yesterday? Yes? No?

If yes, do you use toothpaste? Yes? No?

Dental cost:

During the last year, have you avoided or delayed visiting a dentist because of cost? Yes? No?

How much difficulty would you have paying a $100 dental bill? None, hardly any, a little, a lot

Self-rated health:

Would you rate your general health as: Excellent, very good, good, fair, poor

Would you rate your oral health as: Excellent, very good, good, fair, poor

Dental perceptions:

Do you think you need to have any fillings or extractions? Yes? No?

Do you think you have gum disease? Yes? No?

How often in the last year did you ...

..have toothache?

..feel uncomfortable about the appearance of your teeth, mouth or false teeth?

..have to avoid eating some foods because of problems with your teeth, mouth or false teeth?

Very often, fairly often, occasionally, hardly ever, never

OHIP-14

REALD-30

Oral health knowledge:

How many times do you think teeth should be brushed each day? None, once, twice, more than twice, not sure or don’t know

Should you brush teeth after breakfast? Yes, no, not sure or don’t know

How old should children be when they start to brush their teeth?

Before they have teeth, when their baby teeth first come through, when their big teeth come, not sure or don’t know

Should pre-school children use tooth paste? Yes, no, not sure or don’t know

Are the following things good or bad for teeth or gums? For each item, Good, bad, not sure or don’t know

Fruit, bread, soft-drink, meat, milk, fruit juice, smoking, brushing teeth, bush tucker, lollies/sweets, cordial, water, vegetables, diabetes, toothpaste, visiting a dentist, fluoride.
